# Lobectomy may be more appropriate for patients with early-stage medullary thyroid cancer older than 60 years old

**DOI:** 10.3389/fendo.2022.1015319

**Published:** 2022-10-21

**Authors:** Binfeng Yang, Guangcai Niu, Xiaoxin Li, Fenfen Ma, Yanhong Ma, Shaojun Hu

**Affiliations:** ^1^ Department of Oncology, Suzhou Ninth People’s Hospital, Suzhou, China; ^2^ Department of Gastrointestinal Surgery, Xuzhou Central Hospital, The Affiliated Xuzhou Hospital of Medical College of Southeast University, Xuzhou, China; ^3^ Department of Pathology, Xuzhou Central Hospital, The Affiliated Xuzhou Hospital of Medical College of Southeast University, Xuzhou, China; ^4^ Department of Ultrasound, Xuzhou Central Hospital, The Affiliated Xuzhou Hospital of Medical College of Southeast University, Xuzhou, China; ^5^ Department of Stomatology, Xuzhou Central Hospital, The Affiliated Xuzhou Hospital of Medical College of Southeast University, Xuzhou, China

**Keywords:** medullary thyroid cancer, AJCC, age, total thyroidectomy, lobectomy

## Abstract

**Purpose:**

Clinical guidelines presently recommend total thyroidectomy for the treatment of medullary thyroid cancer (MTC). This study was aimed to investigate whether lobectomy could be the initial treatment for stage I MTC patients.

**Methods:**

The retrospective study was based on data from the Surveillance, Epidemiology, and End Results (SEER) database between 2004 and 2015. The risk factors of survival were estimated by the univariate and multivariate Cox proportional-hazards model. The effect of age on death risk was estimated using restricted cubic splines. Survival curves were constructed according to the Kaplan–Meier method.

**Results:**

A total of 988 stage I MTC patients was included in the study. Among them, 506 (51.2%) MTC patients received lobectomy and 482 (48.8%) received total thyroidectomy. The only independent prognostic factor for overall survival (OS) and disease-specific survival (DSS) was age, according to univariate and multivariate Cox regression analysis. The hazard ratio (HR) increased relatively slowly with age growing under the age of approximately 60 years. However, the death risk of MTC patients began to rise sharply with increasing age above 60 years. For patients under the age of 60, a significant survival difference for OS and DSS was observed between the lobectomy group and total thyroidectomy group (p < 0.05). However, for patients aged above 60, no significant survival difference was observed for OS or DSS (p > 0.05).

**Conclusion:**

Total thyroidectomy was an appropriate treatment for stage I MTC patients under the age of 60, which was consistent with the recommendation of the clinical guidelines. However, for those over the age of 60, lobectomy may be explored as a better surgical option. The findings may provide the evidence base for improving the clinical management of stage I MTC patients. Further prospective multicenter clinical studies are needed including information regarding *RET* status as well as calcitonin and CEA levels.

## Introduction

Thyroid cancer (TC) is the most frequent endocrine neoplasm (1). There are four major pathological types of TC: papillary thyroid cancer (PTC), follicular thyroid cancer (FTC), medullary thyroid cancer (MTC), and anaplastic thyroid cancer (ATC). Unlike the other three types, MTC originates from parafollicular calcitonin secreting cells of the thyroid gland (2), which can produce calcitonin, an important diagnostic and prognostic marker for MTC (3). It accounts for 5%-10% of all TC cases with an annual incidence rate of 0.19/100,000 (4, 5). According to the etiology, MTC could be classified into sporadic MTC and hereditary MTC, accounting for 75% and 25%, respectively (6). *RET* mutations occur in almost all hereditary MTC and a considerable fraction of apparently sporadic MTC cases (7).

Although the 5-year survival rate of early-stage MTC patients is >90%, the overall survival prognosis of MTC patients with all stages is still poor with a 5-year survival rate of less than 40% (8, 9). Therefore, early detection and treatment of MTC are critical. Due to heredity and the lack of effective and safe pharmacotherapy, total thyroidectomy is recommended for treatment of MTC by both the American Thyroid Association (ATA) and British Thyroid Association (BTA) guidelines to prevent cancer recurrence and improve the prognosis (10). However, as compared to lobectomy, total thyroidectomy may increase the risk of postoperative complications such as chronic hypoparathyroidism and laryngeal nerve injury (11). Hence, there have been various attempts to identify a patient subpopulation that would benefit most from minimally invasive surgery, thus reducing the risk of complications (12–14). According to the 2015 ATA guidelines, the initial surgical procedure for patients with solitary intrathyroidal PTC (1-4 cm in size) could be a near-total or total thyroidectomy or lobectomy (12). Mingzhao Xing et al. (15) found that *BRAF* gene status could provide guidance for further optimization of surgical modalities. However, the pros and cons of surgical modalities of MTC patients have been less investigated. Miyauchi et al. (16) analyzed 48 sporadic MTC patients without germline *RET* mutations and found that lobectomy might be an appropriate surgical modality. A single-center, retrospective study from Japan including 118 MTC patients between 1975 and 2005 showed that lobectomy could be considered in sporadic MTC cases with primary tumors located only in one lobe (17). Some scholars suggested that age was an independent prognostic predictor and a less extensive surgical technique might be considered for hereditary MTC patients of younger age (18, 19). These findings remain highly controversial due to small sample sizes or the relatively short follow up period of the referred studies. In the present study, we used the Surveillance, Epidemiology, and End Results (SEER) database to investigate whether lobectomy could be the initial treatment for stage I MTC patients.

## Materials and methods

### Patients

Patient data were extracted from the SEER database between 2004 and 2015. The SEER database is the largest publicly available clinical database covering 18 registries in the United States (20). Patients with MTC were identified using ICD-O-3 codes 8345/3 and 8510/3. Baseline characteristics were collected, including age, race, gender, diagnosis period, grade, and surgical modalities. Inclusion criteria were as follows: (1) patients over 18 years; (2) MTC diagnosis confirmed by histopathological examination; (3) MTC as the only malignancy or the first primary malignancy; (4) stage I (T1N0M0) classification according to the 8^th^ edition of American Joint Committee on Cancer (AJCC) staging system for MTC; and (5) patients underwent lobectomy (surgery codes 20: lobectomy and/or isthmectomy; surgery codes 21: lobectomy only; surgery codes 22: isthmectomy only; surgery codes 23: lobectomy with isthmus) or total thyroidectomy (surgery codes 50: total thyroidectomy). The exclusion criteria were: (1) MTC diagnosis based on radiographic or cytological evidence; (2) no surgical treatment or unclear surgical modalities; and (3) lack of follow-up information. The primary endpoints for survival analysis were overall survival (OS) and disease-specific survival (DSS). OS refers to the time between initial MTC diagnosis and death time from any cause or last follow-up time. DSS refers to the time between initial MTC diagnosis and death time from MTC or last follow-up time. The median follow-up time was 100 months (ranging from 0 to 179 months). The study had been reported in line with the STROCSS criteria (21), and approved by the Medical Scientific Research Ethics Committee of Suzhou Ninth People’s Hospital.

### Statistical analysis

The risk factors of survival were estimated by the univariate and multivariate Cox proportional-hazards model. Hazard ratio (HR) and 95% confidence interval (95% CI) were calculated. Survival curves were constructed according to the Kaplan–Meier method. The effect of age on death risk was estimated using restricted cubic splines. Two-tailed P values of <0.05 indicated a significant difference.

## Results

A total of 988 stage I MTC patients was included in the study (**Table 1**). Age ranged from 18 to 89 years (median: 54). In terms of race, 75.6% of the entire cohort was White, 17.4% was Black and 7.0% was other races. The majority was female, accounting for 85.3%. The percentage of MTC patients was slightly higher during the period 2004-2009 than during 2010-2015. 66 (6.7%) patients had well or moderately differentiated tumors (grade I+II) and 363 (36.7%) had poorly differentiated or undifferentiated tumors (grade III+IV). More than half of the patients (56.6%) had no information about grade. 506 (51.2%) MTC patients underwent lobectomy and 482 (48.8%) underwent total thyroidectomy. The median number of lymph node (LN) examination was 2 in the lobectomy group while that was 3 in the total thyroidectomy group. Patients without LN examination were further excluded and 793 cases were finally included for the following survival analysis.

**Table 1:** Baseline characteristics of stage I MTC patients.

Characteristics	SEER (n=988)
**Age**	
Median (range)	54 (18-89)
**Race**	
White	747 (75.6%)
Black	172 (17.4%)
Others	69 (7.0%)
**Gender**	
Male	145 (14.7%)
Female	843 (85.3%)
**Diagnosis Period**	
2004-2009	515 (52.1%)
2010-2015	473 (47.9%)
**Grade**	
I+II	66 (6.7%)
III+IV	363 (36.7%)
Unknown	559 (56.6%)
**Surgical Intervention**	
Lobectomy	506 (51.2%)
Total Thyroidectomy	482 (48.8%)
**No. of LN examination, Median (range)**	
Lobectomy	3 (0-37)
Total Thyroidectomy	2 (0-84)
**Percentage of LN examination**	
Lobectomy	89.1%
Total Thyroidectomy	71.0%

SEER, Surveillance Epidemiology and End Results; MTC, medullary thyroid cancer.

**Figure 1:**
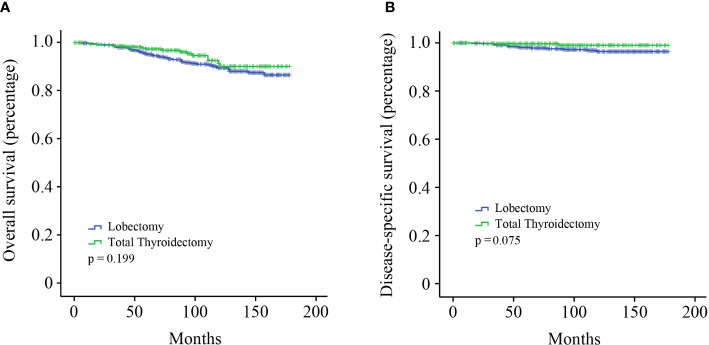
Kaplan–Meier survival analysis. **(A)** OS curve of stage I MTC patients; **(B)** DSS curve of stage I MTC patients.

The cohort was categorized into two groups to investigate the effect of different surgical modalities on stage I MTC patients: lobectomy and total thyroidectomy. Kaplan–Meier survival analysis showed that no significant survival difference for OS was observed between the two groups (**Figure 1A**, p = 0.199). DSS in patients who underwent total thyroidectomy seemed to be better than those undergone lobectomy, although not reaching statistical significance (**Figure 1B**, p = 0.075).

**Figure 2:**
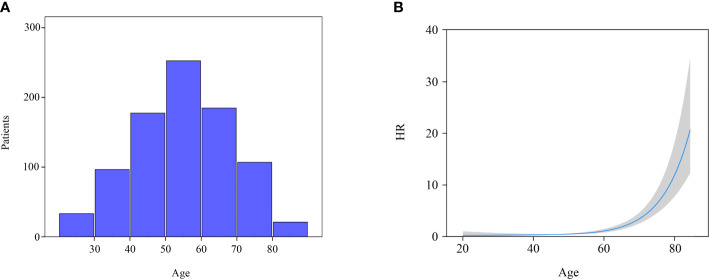
Distribution and effect of age. **(A)** Age distribution of entire cohort; **(B)** Influence of age on the death risk of stage I MTC patients by means of restricted cubic splines.

**Figure 3:**
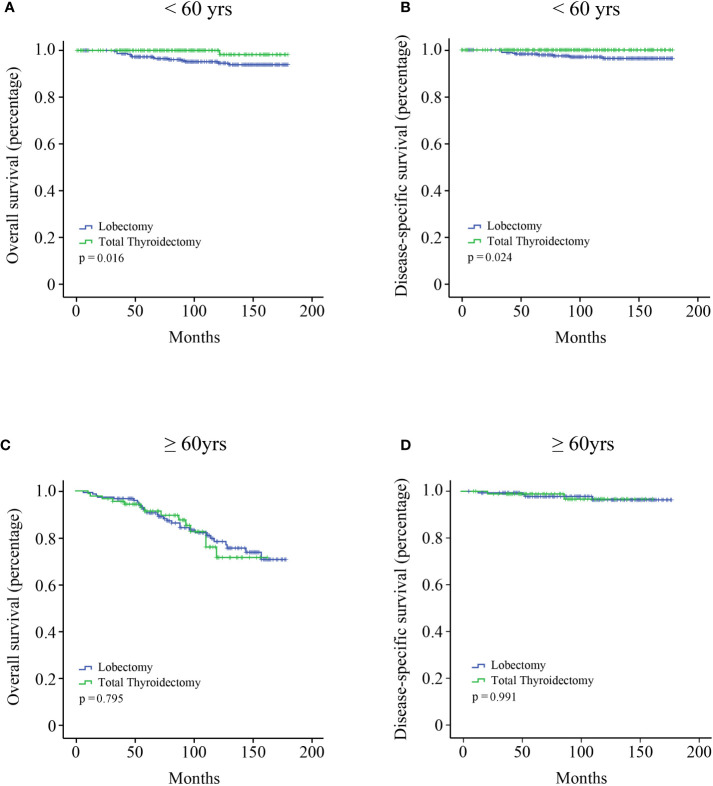
Kaplan–Meier survival analysis according to the age subgroups. **(A)** OS curve of stage I MTC patients under the age of 60 years; **(B)** DSS curve of stage I MTC patients under the age of 60 years; **(C)** OS curve of stage I MTC patients above the age of 60 years; **(D)** DSS curve of stage I MTC patients above the age of 60 years.

All the variables were included to perform univariate and multivariate Cox regression analysis (**Table 2**). Age is a continuous variable, and we investigated the optimal age cutoff value to transform age into a categorical variable. As shown in **Figure 2A**, the distribution of age followed a normal distribution, and the age group 50-60 years had the highest percentage (29.8%). The HR increased relatively slowly with age growing under the age of approximately 60 years (**Figure 2B**). However, the risk of mortality in MTC patients began to rise sharply with increasing age above 60 years. Thus, the age of 60 years was considered as the optimal cutoff value. Both univariate and multivariate analyses showed that age was the only independent prognostic factor. Given this, the two groups (lobectomy and total thyroidectomy) were further separated into age-related subgroups. There were 505 cases under the age of 60 and 288 cases over 60. The median follow-up time of patients under the age of 60 was 105 months while that of patients over 60 was 90 months. For patients under the age of 60, a significant survival difference for OS and DSS was observed between the lobectomy group and total thyroidectomy group (**Figures 3A, B**, p < 0.05). For patients aged above 60, however, no significant survival difference was observed for OS or DSS (**Figures 3C, D**).

**Table 2:** Univariate and multivariate Cox regression analysis of OS in stage I MTC patients.

Characteristics	Univariate Analysis		Multivariate Analysis	
	HR (95%CI)	P value	HR (95%CI)	P value
**Age**				
<60	Reference		Reference	
≥60	7.250 (4.452-11.808)	<0.001	7.275 (4.459-11.870)	<0.001
**Race**				
White	Reference		Reference	
Black	0.838 (0.488-1.439)	0.522	0.849 (0.486-1.483)	0.565
Others	0.332 (0.081-1.352)	0.124	0.324 (0.079-1.331)	0.118
**Gender**				
Male	Reference		Reference	
Female	0.926 (0.515-1.666)	0.797	1.007 (0.528-1.920)	0.983
**Diagnosis Period**				
2004-2009	Reference		Reference	
2010-2015	0.794 (0.474-1.329)	0.380	0.742 (0.436-1.263)	0.272
**Grade**				
I+II	Reference		Reference	
III+IV	0.734 (0.327-1.651)	0.455	0.879 (0.383-2.015)	0.761
Unknown	0.721 (0.326-1.595)	0.420	0.839 (0.359-1.962)	0.686
**No. of LN examination**	0.988 (0.970-1.007)	0.224	0.943 (0.931-0.955)	0.206
**Surgical Intervention**				
Lobectomy	Reference		Reference	
Total Thyroidectomy	0.906 (0.595-1.378)	0.644	0.924 (0.509-1.677)	0.796

CI, confidence interval; HR, hazard ratio; OS, overall survival; MTC, medullary thyroid cancer.

## Discussion

Early-stage MTC patients usually have a favorable prognosis. It is still unknown whether there is a subset of early MTC patients that are overtreated based on the commonly used clinical guidelines. Using the population-based database, it was found that total thyroidectomy was an appropriate treatment for stage I MTC patients under the age of 60. However, for those older than 60 years of age, lobectomy may be considered as a better surgical procedure since total thyroidectomy could not provide an additional survival benefit. The less extensive operation would reduce the incidence of complications and improve the quality of life. These findings may provide some guidance for clinical decisions.

Age was reported to be an independent prognostic factor for MTC patients in various large sample studies (18, 22, 23). A study by Sahli et al. (18) classified MTC patients into three groups (18-64 years, 65-79 years, and ≥ 80 years) based on the previous studies of PTC and found that elderly patients (≥ 65 years) had a greater risk of mortality than young patients (with age group 18-64 years as reference, HR: 1.787). MTC patients were also classified into three groups (18-49 years, 50-69 years, and ≥ 70 years) in the study by Qu et al. (24) With age group 18-49 years as reference, the HR of age group 50-69 years and ≥ 70 years were 2.853 and 5.804, respectively. In the present study, we investigated the effect of age on the risk of mortality using restricted cubic splines and found that the survival curve was anti-L-shaped. The HR was virtually unchanged under the age of 60 years and began to rise sharply above 60 years, indicating it was justified to classify these MTC patients into two groups rather than three groups. Our results also demonstrated that the HR of elderly patients (≥ 60 years) was significantly higher compared to elderly group in the research by Sahli et al. and Qu et al. (with age group <60 years as reference, HR: 7.275).

MTC tumors in elderly patients (≥ 60 years) tend to present with more aggressive biological behavior and higher lethality. Generally, more extensive and radical surgery is needed for tumors with more aggressive histology. However, our findings revealed that elderly patients (≥ 60 years) did not benefit from more extensive surgery (total thyroidectomy) while young patients did. This might be explained by the fact that the age factor played a much more important role in elderly patients than surgery. Whatever surgical procedures were used, the clinical outcomes of elderly patients could not be improved. The findings provided a foundation for improving the ATA recommendations. For young patients (< 60 years) whose prognosis was hardly affected by the age factor, total thyroidectomy could significantly improve the clinical outcomes. These findings were consistent with the recommendation of the ATA guidelines. Sahli et al. (18) also found that young patients underwent total thyroidectomy at a higher proportion compared to elderly patients by analyzing MTC patients with all stages in the SEER database.

LN metastasis of MTC is common (25), and the ATA guidelines recommended that central LN dissection should be performed regardless of LN status (10). There is currently a disagreement over lateral LN dissection (26–28). However, Hamy et al. (29) showed that early MTC tumors (T1a) had almost no LN metastatic potential, suggesting LN dissection may not be suitable for early-stage MTC. Niederle et al. (30) indicated that MTC desmoplastic stromal reaction negative (DSR-negative) tumors generally had a small diameter (T1a, <1cm) and no lateral LN metastasis. Lateral LN dissection was not necessary for individuals with DSR-negative tumors. Our study also demonstrated that the number of LN examination was not an independent prognostic factor for stage I MTC. Nonetheless, the SEER database did not distinguish the extent and location of LN examination (central or lateral), and definitive and reliable conclusions could not be reached.

There are several limitations in the present study. First, it is the well-known limitation of the retrospective nature of the SEER database. Further prospective, multicenter clinical trials should be performed to validate our results. Second, the *RET* status, CEA or calcitonin levels were not recorded in the SEER database. A more comprehensive multivariate Cox regression analysis could not be carried out. Third, stage I MTC patients have a comparatively favorable outcome and statistically significant survival differences are difficult to be recorded. There might be differences in OS between the lobectomy and total thyroidectomy groups for patients under the age of 60 regarding longer follow-up time and larger cohort. Fourth, the decision about the appropriate surgical approaches could not only be dependent on OS and DSS but also the postoperative complications and the quality of life. Due to a lack of information about postoperative complications and recurrence, a more accurate and comprehensive assessment could not be conducted. Fifth, in 56.6% of stage I MTC patients information about the grade in the SEER database were not available. Thus, it was not able to identify whether grade was an independent prognostic factor for stage I MTC patients. Studies indicated that grade was significantly associated with survival outcomes of MTC with all stages (31, 32).

In summary, total thyroidectomy was an appropriate treatment for stage I MTC patients under the age of 60, which was consistent with the recommendation of the clinical guidelines. However, for those over the age of 60, lobectomy may be considered as a better surgical option. The findings may provide the evidence base for improving the clinical management of stage I MTC patients. Further prospective multicenter clinical studies are needed including information regarding *RET* status as well as calcitonin and CEA levels.

## Data availability statement

Publicly available datasets were analyzed in this study. This data can be found here: https://seer.cancer.gov/.

## Ethics statement

The studies involving human participants were reviewed and approved by Medical Scientific Research Ethics Committee of Suzhou Ninth People’s Hospital. The patients/participants provided their written informed consent in the SEER database.

## Author contributions

BY and SH made substantial contributions to the design of the study, carried out the analysis, interpreted the data. GN and XL contributed to the review of previous literature. FM and YM contributed substantially to the data discussion and critically commented on the manuscript for scientific content. All authors made substantial contributions to the conception and design of the study, data interpretation and drafting of the manuscript, were responsible for the quality of the overall manuscript. All authors approved the final version of the manuscript.

## Acknowledgments

The authors are thankful to the statistician Aidibai Simayi from Department of Epidemiology and Health Statistics, School of Public Health, Southeast University.

## Conflict of interest

The authors declare that the research was conducted in the absence of any commercial or financial relationships that could be construed as a potential conflict of interest.

## Publisher’s note

All claims expressed in this article are solely those of the authors and do not necessarily represent those of their affiliated organizations, or those of the publisher, the editors and the reviewers. Any product that may be evaluated in this article, or claim that may be made by its manufacturer, is not guaranteed or endorsed by the publisher.
