# Corrigendum: Lobectomy may be more appropriate for patients with early-stage medullary thyroid cancer older than 60 years old

**DOI:** 10.3389/fendo.2022.1111155

**Published:** 2022-12-07

**Authors:** Binfeng Yang, Guangcai Niu, Xiaoxin Li, Fenfen Ma, Yanhong Ma, Shaojun Hu

**Affiliations:** ^1^ Department of Oncology, Suzhou Ninth People’s Hospital, Suzhou, China; ^2^ Department of Gastrointestinal Surgery, Xuzhou Central Hospital, The Affiliated Xuzhou Hospital of Medical College of Southeast University, Xuzhou, China; ^3^ Department of Pathology, Xuzhou Central Hospital, The Affiliated Xuzhou Hospital of Medical College of Southeast University, Xuzhou, China; ^4^ Department of Ultrasound, Xuzhou Central Hospital, The Affiliated Xuzhou Hospital of Medical College of Southeast University, Xuzhou, China; ^5^ Department of Stomatology, Xuzhou Central Hospital, The Affiliated Xuzhou Hospital of Medical College of Southeast University, Xuzhou, China

**Keywords:** medullary thyroid cancer, AJCC, age, total thyroidectomy, lobectomy

## Incorrect Author Name

In the published article, an author name was incorrectly written as Gaungcai Niu. The correct spelling is Guangcai Niu.

The authors apologize for this error and state that this does not change the scientific conclusions of the article in any way. The original article has been updated.

